# Computed tomography assessment of body composition in patients with nonmetastatic breast cancer: what are the best prognostic markers?

**DOI:** 10.1590/0100-3984.2022.0022

**Published:** 2022

**Authors:** José Carlos Oliveira Júnior, Thais Manfrinato Miola, Stefânia Maria Roman, Humberto Oliart-Guzmán, Vinícius Silva Oliveira, Juliana de Oliveira Souza, Fabiana Baroni Makdissi, Almir Galvão Vieira Bitencourt

**Affiliations:** 1 A.C.Camargo Cancer Center, São Paulo, SP, Brazil.

**Keywords:** Breast neoplasms, Tomography, X-ray computed, Body composition, Prognosis, Neoplasias da mama, Tomografia computadorizada, Composição corporal, Prognóstico

## Abstract

**Objective:**

To correlate body composition measures, based on computed tomography (CT) analysis of muscle mass and adipose tissue, with disease-free survival in breast cancer patients.

**Materials and Methods:**

This single-center retrospective study included 262 female patients with nonmetastatic breast cancer. Body composition was assessed on a pretreatment CT scan (at the L3 level). The analysis included quantification of the areas of subcutaneous adipose tissue (SAT), visceral adipose tissue (VAT), and skeletal muscle mass, as well as of the mean skeletal muscle density. The VAT/SAT ratio, skeletal mass index (SMI), and skeletal muscle gauge (SMG) were calculated.

**Results:**

Of the 262 patients evaluated, 175 (66.8%) were classified as overweight or obese on the basis of their body mass index. We observed low SMI in 35 patients (13.4%) and elevated VAT in 123 (46.9%). Disease-free survival was significantly shorter in the patients who underwent neoadjuvant chemotherapy (*p* = 0.044), in those with a low SMI (*p* = 0.006), in those with low SMG (*p* = 0.013), and in those with a low VAT/SAT ratio (*p* = 0.050). In a multivariate analysis, only SMG, the VAT/SAT ratio, and having undergone neoadjuvant chemotherapy retained their statistical significance.

**Conclusion:**

Our results confirm that low SMG and the VAT/SAT ratio can be used as imaging biomarkers to assess prognosis in patients with nonmetastatic breast cancer.

## Abstract (pt)

**Objetivo:**

Correlacionar medidas de composição corporal baseadas na análise por tomografia computadorizada (TC) da massa muscular e tecido adiposo com a sobrevida livre de doença em pacientes com câncer de mama.

**Materiais e Métodos:**

Estudo retrospectivo unicêntrico que incluiu 262 mulheres com câncer de mama não metastático. Avaliação da composição corporal foi realizada na TC pré-tratamento (nível de L3), incluindo tecido adiposo subcutâneo (SAT) e tecido adiposo visceral (VAT), massa muscular esquelética e densidade do músculo esquelético. A relação VAT/SAT, o índice de massa esquelética (SMI) e o produto do músculo esquelético (SMG) foram calculados.

**Resultados:**

De acordo com o índice de massa corporal, 175 (66,8%) pacientes foram classificadas com sobrepeso ou obesidade. Baixo SMI foi observado em 35 (13,4%) pacientes e VAT elevado, em 123 (46,9%). Houve uma redução significativa na sobrevida livre de doença nas pacientes que realizaram quimioterapia neoadjuvante (*p* = 0,044), nas com baixo SMI (*p* = 0,006), baixo SMG (*p* = 0,013) e baixa relação VAT/SAT (*p* = 0,050). Na análise multivariada, apenas SMG, relação VAT/SAT e quimioterapia neoadjuvante confirmaram significância estatística.

**Conclusão:**

Nossos resultados confirmaram que SMG e relação VAT/SAT baixos podem ser usados como importantes biomarcadores de imagem para avaliar o prognóstico em pacientes com câncer de mama não metastático.

## INTRODUCTION

Nutritional status and body composition parameters are important factors in breast cancer treatment^(1)^. Obesity is a known risk factor for breast cancer development, especially after menopause, and is associated with a poorer prognosis in breast cancer patients^(2,3)^. Recently, sarcopenia (low muscle mass) has also proven to be a major risk factor for mortality among breast cancer patients^(4)^.

Computed tomography (CT) is considered the gold standard for body composition assessment in oncology^(5)^, including the analysis of skeletal muscle mass (SMM), subcutaneous adipose tissue (SAT), and visceral adipose tissue (VAT). Most cancer patients frequently undergo CT for diagnosis, staging, and evaluation of treatment response; those same examinations can be used in order to assess body composition without additional doses of radiation^(6)^.

Various CT-based muscle mass and adipose tissue measures have been found to correlate with the breast cancer prognosis^(7,8,9,10,11,12,13,14,15)^. However, there is still controversy regarding the best CT body composition biomarker to predict outcomes. The aim of this study was to determine whether body composition measures based on CT analysis of SMM, SAT, and VAT correlate with disease-free survival (DFS) in patients newly diagnosed with nonmetastatic breast cancer.

## MATERIALS AND METHODS

This was a single-center, retrospective cohort study including female patients newly diagnosed with nonmetastatic breast cancer between January 2016 and January 2018 at a referral cancer center in the city of São Paulo, Brazil. Patients for whom pretreatment abdominal CT images were not available for analysis were excluded, as were those who did not complete the treatment and follow-up at the same center. The study was approved by the local institutional review board.

Clinical information was obtained from electronic medical records, including patient age, weight, height, tumor size, clinical staging, histological type, molecular subtype, treatment (surgery, chemotherapy, radiation, and hormone therapy), and outcome during follow-up (recurrence and death). For each patient, the body mass index (BMI) was calculated as weight in kilograms divided by the square of height in meters. Clinical staging was assessed by using the eighth edition of the American Joint Committee on Cancer tumor–node–metastasis staging system. All pretreatment biopsies were reviewed by the pathology department of the institution. Tumor histological types were reported according to the World Health Organization classifi cation of tumors and molecular subtypes and the St. Gallen criteria^(16)^. For patients submitted to neoadjuvant chemotherapy (NAC), the pathological response was assessed according to the residual cancer burden protocol^(17)^.

**Figure 1:**
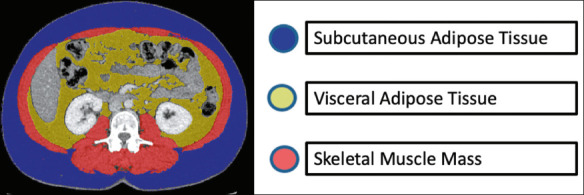
Example of an axial tomographic slice acquired at the L3 level, showing the surface areas of SAT, VAT, and SMM.

All CT examinations were performed in a multidetector scanner, and all of the resulting images were reviewed by the same radiologist. Body composition was assessed by analyzing an axial CT slice acquired at the level of the third lumbar vertebral body (L3). We assessed the surface areas of SAT, VAT, and SMM by a semi-automatic method with manual correction^(18)^, using the CoreSlicer software package (https://coreslicer.com/), as depicted in Figure 1. Adipose tissue was defi ned as tissue with a density from −190 to −30 HU, and muscle mass was defi ned as tissue with a density from −29 to +150 HU. Elevated VAT was defi ned as a VAT area ≥ 100 cm^2^^(19)^. In addition, the VAT/SAT ratio was calculated as previously proposed^(12)^. The skeletal mass index (SMI) was defi ned as the area of muscle mass divided by square of height, and muscle mass depletion was defi ned as an SMI ≤ 39 cm^2^/m^2^^(20)^. The mean skeletal muscle density (SMD, in HU) was also assessed, and skeletal muscle gauge (SMG) was determined by multiplying the SMI by the SMD^(7)^. Finally, to assess the morphology of the psoas muscle, the long and short axes of the muscle were measured in the same axial CT slice at the L3 level. The morphology of the psoas muscle was defi ned as the ratio between the short and long axes and graded as follows^(21)^: > 2/3 (grade 0); ≤ 2/3 and > 1/2 (grade 1); ≤ 1/2 and > 1/3 (grade 2); ≤ 1/3 and > 1/4 (grade 3); and < 1/4 (grade 4).

The statistical analysis was performed with the IBM SPSS Statistics software package for Windows, version 20.0 (IBM Corp., Armonk, NY, USA). Categorical variables were expressed as absolute and relative frequencies, whereas quantitative variables were expressed as range, mean, and standard deviation or as median and interquartile range for those with non-normal distribution. Chi-square and Fisher’s exact tests were used in order to compare categorical variables; Student’s t-tests or nonparametric Mann-Whitney tests were used to compare quantitative variables between two groups according to the variable distribution. Kaplan-Meier curves were used in order to analyze DFS. The log-rank test and simple Cox regression were used in order to compare the survival curves between groups, as well as to estimate the hazard ratios and 95% confidence intervals, respectively. Continuous variables with no well-established cutoff values (such as SMG and the VAT/SAT ratio) were stratified into two groups by using a cutoff point that was estimated by the maximally selected standardized log-rank statistic method. The estimated cutoffs were 0.47 for the VAT/SAT ratio and 1,666 for SMG. For the multivariate analysis, multiple Cox regression models were fitted for variables that achieved a *p* ≤ 0.1 in the univariate Cox regression analysis; the final model was obtained using the backward stepwise (likelihood ratio) method. The level of significance adopted was 5% (*p* ≤ 0.05).

## RESULTS

A total of 375 patients met the inclusion criteria. Of those, 113 were excluded because there were no CT images available for analysis. Therefore, 262 patients were included. The mean age of the patients in the sample was 51.9 ± 12.4 years (range, 27–86 years). The clinical characteristics and treatment data are shown in Table 1. The mean BMI was 27.4 ± 5.1 kg/m^2^ (range, 13.8–46.3 kg/ m^2^), two patients (0.8%) being classified as underweight, 85 (32.4%) being classified as normal weight, 108 (41.2%) being classified as overweight, and 67 (25.6%) being classified as obese.

**Table 1:** Clinical characteristics of female patients newly diagnosed with nonmetastatic breast cancer and characteristics of the treatment received by those patients.

Variable	(N = 262)
T staging, n (%)	
T1	83 (31.7)
T2	105 (40.1)
T3	50 (19.1)
T4	24 (9.2)
N staging, n (%)	
N1	128 (48.9)
N2	84 (32.1)
N3	37 (14.1)
N4	13 (5.0)
Clinical stage, n (%)	
I	69 (26.3)
II	101 (38.5)
III	92 (35.1)
Histological type, n (%)	
No special type (invasive ductal carcinoma)	217 (82.8)
Special types	44 (16.8)
Molecular subtype, n (%)	
Luminal A	41 (15.6)
Luminal B	143 (54.6)
HER2	35 (13.4)
Triple-negative	41 (15.6)
NAC, n (%)	100 (38.2)
Response to NAC, n (%)	
RCB 0 (complete response)	36 (36.7)
RCB I	7 (7.1)
RCB II	32 (32.7)
RCB III	23 (23.5)
Surgery, n (%)	
Conservative	83 (31.7)
Mastectomy	178 (67.9)
Adjuvant chemotherapy, n (%)	169 (64.5)
Adjuvant radiation therapy, n (%)	212 (80.9)
Hormone therapy, n (%)	205 (78.2)

A descriptive analysis of CT body composition measures is shown in Table 2. A low SMI was observed in 35 patients (13.4%), and elevated VAT was observed in 123 (46.9%). The morphology of the psoas muscle was classified as grade 0 in 35 patients (13.4%), grade 1 in 127 (48.5%), grade 2 in 97 (37.0%), grade 3 in two (0.8%), and grade 4 in one (0.4%).

**Table 2:** Descriptive analysis of CT body composition measures in female patients newly diagnosed with nonmetastatic breast cancer.

Variable	Mean ± standard deviation (range)
SMM	118.6 ± 19.6 cm^2^ (12.1–188.5 cm^2^)
SMI	45.9 ± 7.2 cm^2^/m^2^ (4.2–71.9 cm^2^/m^2^)
SMD	36.9 ± 11.1 HU (6.0–127.9 HU)
SMG	1691.6 ± 559.2 HU cm^2^/m^2^ (100.8–6163.0 HU cm^2^/m^2^)
VAT	106.5 ± 74.0 cm^2^ (8.0–353.8 cm^2^)
S AT	238.7 ± 114.1 cm^2^ (24.8–818.7 cm^2^)
VAT/SAT ratio	0.45 ± 0.28 (0.07–1.79)

**Figure 2:**
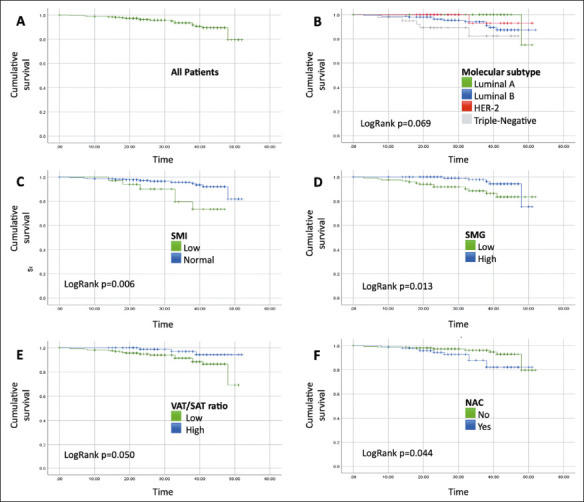
Kaplan-Meier survival curve analyses of breast cancer patients, overall (A) as well as by molecular subtype (B), SMI (C), SMG (D), VAT/SAT ratio (E), and history of NAC (F). HER2, human epidermal growth factor receptor 2.

The mean duration of follow-up was 32.8 ± 1.8 months, with a median of 33 months (interquartile range, 29.5–36.5 months). During follow-up, 11 patients (4.2%) had local recurrence, 27 (10.3%) had distant metastasis, and seven (2.7%) died. In the univariate analysis, the variables that showed a significant association with recurrence were the SMI, SMG, VAT/SAT ratio, having the triple-negative breast cancer subtype, and having undergone NAC (Table 3). As illustrated in Figure 2, Kaplan-Meyer curves also showed that DFS was significantly shorter in the patients who underwent NAC (*p* = 0.044), in those with a low SMI (*p* = 0.006), in those with low SMG (*p* = 0.013), and in those with a high VAT/SAT ratio (*p* = 0.050). In the multivariate analysis, only SMG, the VAT/SAT ratio, and having undergone NAC retained their statistical significance (Table 4).

**Table 3:** Univariate Cox regression of DFS in female patients with nonmetastatic breast cancer according to demographic, CT-based, and clinical body composition measures.

Variable	Categories	Coeffcient	SE	HR	95% CI	*P*
Age	≥ 50 years			Reference		
	< 50 years	–0.274	0.476	0.760	0.299–1.932	0.565
SMI	Normal			Reference		
	Low	1.303	0.508	3.682	1.361–9.961	0.010
SMG	High			Reference		
Low	1.228	0.527	3.416	1.217–9.589	0.020
VAT/SAT ratio	Low			Reference		
High	–1.170	0.633	0.310	0.090–1.073	0.064
VAT	Elevated			Reference		
Normal	0.672	0.501	1.959	0.734–5.225	0.179
Histological type	Special types			Reference		
No special type	–0.193	0.568	0.824	0.271–2.507	0.733
Clinical staging	I			Reference		
	II	1.276	0.792	3.583	0.759–16.921	0.107
	III	1.363	0.793	3.910	0.827–18.489	0.085
Molecular subtype	Luminal A			Reference		
	Luminal B	1.392	1.057	4.024	0.507–31.940	0.188
	HER2	1.416	0.080	1.491	0.093–23.920	0.778
	Triple-negative	2.236	1.119	9.358	1.043–83.960	0.046
NAC	No			Reference		
	Yes	0.926	0.477	2.252	0.992–6.430	0.052
Surgery type	Mastectomy			Reference		
	Breast-conserving	–0.132	0.501	0.876	0.328–2.342	0.792

SE, standard error; HR, hazard ratio; CI, confidence interval; HER2, human epidermal growth factor receptor 2.

**Table 4:** Multiple Cox regression of DFS in female patients with nonmetastatic breast cancer according to CT-based and clinical body composition measures.

Variable	Category	Coeffcient	SE	HR	95% CI	*P*
SMG	Low	1.601	0.537	4.956	1.728–14.211	0.003
VAT/SAT ratio	Low	1.478	0.647	4.386	1.234–15.583	0.022
NAC	Yes	0.953	0.482	2.593	1.008–6.668	0.048

SE, standard error; HR, hazard ratio; CI, confidence interval;

## DISCUSSION

Our results show that body composition analysis by CT has considerable prognostic value in nonmetastatic breast cancer. Measures of muscle mass were found to be related to DFS, as were measures of adipose tissue.

Caan et al.^(11)^ evaluated 3,241 patients with stage II or III breast cancer and found that 34% presented with a low SMI. Using the same criteria applied by those authors, we found the prevalence of low SMI to be only 13% in the present study, which could be explained by the differences between their sample and ours, in which 26% of the patients had stage I breast cancer. In the Caan et al.^(11)^ study, sarcopenia and adiposity from clinically acquired CT scans both provided significant prognostic information that outperforms BMI.

Results in the literature vary regarding the impact of sarcopenia on the prognosis of nonmetastatic breast cancer. In a systematic review conducted by Rossi et al.^(8)^, the authors identified 13 studies evaluating the impact that sarcopenia assessed by CT (at the L3 level) has on clinical outcomes. Among the studies of this topic, eight concluded that sarcopenia is a major risk factor for a poor prognosis in breast cancer and five found no significant association between the two.

Most of the relevant studies in the literature define sarcopenia as a low SMI on CT. However, that definition is outdated. As defined by the European Working Group on Sarcopenia in Older People, sarcopenia is a progressive, generalized skeletal muscle disorder associated with an increased likelihood of adverse outcomes. In its 2019 definition, the Group used low muscle strength as the primary parameter of sarcopenia, while recommending that the presence of low muscle quantity or poor muscle quality be used in order to confirm the diagnosis^(22)^. The SMI has long been used as a measure of muscle quantity, and radiodensity on CT has recently been proposed as a measure of muscle quality^(23)^. The SMD conveys the composition of muscle tissue, independent of muscle quantity, and is inversely related to fatty infiltration of skeletal muscle, known as myosteatosis. The SMI and SMD are defined independently of one another, and both are demonstrated prognostic indicators for cancer outcomes. A meta-analysis conducted by Aleixo et al.^(15)^ showed that sarcopenia is associated with greater chemotherapy toxicity as well as shorter survival among women with early-stage nonmetastatic breast cancer, and that low muscle density is prognostic of overall survival in metastatic breast cancer.

Weinberg et al.^(7)^ suggested the use of SMG as a new metric to provide an integrated measure of the quality and quantity of skeletal muscle. The authors evaluated 241 patients with early-stage breast cancer and found that SMG correlated better with increasing age than did the SMI or SMD alone, although they did not explore its impact on outcomes. In the multivariate analysis performed in the present study, SMG was found to be a better predictor of DFS than was the SMI, suggesting that the use of SMG as a metric could improve the evaluation of skeletal muscle by CT.

The results of the present study also show that DFS was shorter among the patients with a low VAT/SAT ratio. Deluche et al.^(12)^ evaluated 119 women with early-stage breast cancer and found that a lower VAT/SAT ratio was associated with shorter DFS and shorter overall survival in the univariate analysis but not in the multivariate analysis, probably because of their small sample size. Bradshaw et al.^(9)^ assessed the relationships that VAT and SAT had with survival among 3,235 women with stage II or III breast cancer. They found that SAT was related to an increased risk of death, although they found no such relationship for VAT. Those authors suggested that SAT is an underappreciated risk factor for breast cancer-related death.

The factors related to a worse prognosis in patients with a low VAT/SAT ratio are not yet fully understood. Although VAT is often cited as the relevant measure because of its systemic effects on insulin resistance, infl ammation, and endogenous estrogen synthesis^(24)^, abdominal SAT may have metabolic effects similar to, and independent from, those of VAT^(25)^. Abdominal SAT is also more strongly correlated with breast adipose tissue than is VAT^(26,27)^. Breast adipose tissue is involved in the production of inflammatory cytokines and promotes endogenous estrogen production. Therefore, a relative increase in SAT in relation to VAT (i.e., a lower VAT/SAT ratio) could be associated with greater inflammation of breast adipose tissue, which provides an environment thought to encourage tumor growth and development^(28)^.

Our study has some limitations, primarily related to its retrospective design, the heterogeneity of our sample, and the relatively short follow-up period. Because abdominal CT scans are not systematically used for staging in all breast cancer patients, we included only patients who had an initial CT scan based on institutional protocols.

In conclusion, our results confirm that CT-based body composition measures could be used as important imaging biomarkers to assess prognosis in patients with nonmetastatic breast cancer. Low SMG and a low VAT/SAT ratio appear to be independently associated with worse DFS in populations such as the one evaluated here. We believe that analysis of muscle mass should be incorporated into the routine assessment of breast cancer patients who will undergo CT for other reasons (e.g., staging or response evaluation), in order to provide additional useful information to guide therapy.
